# Development of a Machine Learning‑Based Prognostic Model for Intermediate Trophoblastic Tumors: A Single-Center Study With Web-Based Tool Implementation

**DOI:** 10.1200/PO-25-00881

**Published:** 2026-06-12

**Authors:** Weidi Wang, Yunshu Jiao, Yuan Li, Fang Jiang, Xirun Wan, Fengzhi Feng, Jun Zhao, Tong Ren, Dan Wang, Ming Du, Chen Li, Zhen Zheng, Duancheng Tian, Junjun Yang, Yang Xiang

**Affiliations:** ^1^ National Clinical Research Center for Women’s Health and Obstetric and Gynecologic Diseases, Department of Obstetrics and Gynecology, Peking Union Medical College Hospital, Chinese Academy of Medical Sciences & Peking Union Medical College, Beijing, China; ^2^ Department of Clinical Medicine, Chinese Academy of Medical Sciences & Peking Union Medical College, Beijing, China

## Abstract

**PURPOSE:**

Current prognostic systems are inadequate for intermediate trophoblastic tumors (ITTs). The aim of this study was to develop a machine learning (ML)-based model to predict progression-free survival (PFS) in patients with ITT and implement a web-based tool for individualized risk stratification.

**MATERIALS AND METHODS:**

We analyzed a retrospective cohort of 236 patients with ITT treated at a national tertiary center between 2000 and 2024. A multimodal feature selection strategy—integrating Cox regression, LASSO, Gradient Boosting Machine (GBM), and Random Survival Forest (RSF)—was used to identify robust predictors from clinicopathologic and inflammatory variables. The final prognostic model was constructed using the RSF approach. Model performance was evaluated through a rigorous nested 5-fold cross-validation framework to prevent data leakage.

**RESULTS:**

Five key predictors were identified: International Federation of Gynecology and Obstetrics stage, interval from antecedent pregnancy, Ki-67 index, neutrophil-to-lymphocyte ratio, and systemic immune-inflammation index. The RSF model demonstrated robust discrimination with a cross-validated concordance index of 0.816 (95% CI, 0.721 to 0.895) and excellent calibration (Integrated Brier Score, 0.113). Decision-curve analysis confirmed clinical utility within the relevant 20%-60% threshold range. An interactive web tool (Shinyapps) was deployed to generate real-time individualized PFS predictions with 95% confidence intervals.

**CONCLUSION:**

To our knowledge, this study presents the first ML-based prognostic model specifically for ITT. By integrating immune-inflammatory markers with traditional clinicopathologic features, the RSF model offers superior risk stratification compared with anatomic staging or alternative models. The developed online tool serves as a proof-of-concept prototype to facilitate future external validation and research on personalized clinical decision making for this rare malignancy.

## INTRODUCTION

Gestational trophoblastic neoplasia (GTN) represents a group of malignancies arising from abnormal proliferation of placental trophoblastic cells, encompassing invasive hydatidiform mole, choriocarcinoma, placental site trophoblastic tumor (PSTT), and epithelioid trophoblastic tumor (ETT). Among these, PSTT and ETT together account for only approximately 1%-3% of all patient cases with GTN^1^; both originate from extravillous trophoblastic cells and are composed of intermediate trophoblasts, collectively termed intermediate trophoblastic tumors (ITTs).^2^ Despite certain histopathologic distinctions, they share significant clinical and biologic similarities.^3^

**CONTEXT**

**Key Objective**
This study develops and validates the first machine learning–based prognostic model for intermediate trophoblastic tumors, using a rigorous nested cross-validation framework to overcome the limitations of traditional staging systems in rare malignancies.
**Knowledge Generated**
A robust five-factor signature comprising International Federation of Gynecology and Obstetrics stage, interval from antecedent pregnancy, Ki-67 index, neutrophil-to-lymphocyte ratio, and systemic immune-inflammation index was identified through multimodal feature selection. The Random Survival Forest model demonstrated superior discrimination (C-index, 0.816) and calibration compared with anatomic staging or alternative models.
**Relevance**
The deployed web-based calculator provides a transparent prototype for real-time risk stratification. Pending independent external validation, it holds the potential to facilitate personalized treatment escalation or de-escalation strategies for this rare disease.

Compared with choriocarcinoma, ITTs exhibit relatively indolent growth, lower human chorionic gonadotropin (HCG) secretion, and chemoresistance, rendering the International Federation of Gynecology and Obstetrics (FIGO) prognostic scoring system inadequate for guiding their clinical management.^2^ Current guidelines^2,3^ rely on high-risk factors such as advanced FIGO stage and interval from antecedent pregnancy (IFAP) ≥48 months to guide clinical decision making. Nevertheless, significant prognostic heterogeneity persists, with recurrence rates of 8.8%-13.3% in early-stage patients^4-6^ and long-term survival achieved in 42%-65% of advanced patient cases.^4,7^

The peripheral immune-inflammatory response, quantified through markers like neutrophil-to-lymphocyte ratio (NLR) and systemic immune-inflammation index (SIRI), has emerged as a crucial mediator of tumor microenvironment interactions and metastatic progression across multiple malignancies.^8^ Although these parameters have demonstrated robust prognostic value in various cancers,^9-11^ their clinical utility in ITT remains unexplored, primarily because of disease rarity.

Contemporary machine learning (ML) methodologies, such as random survival forests (RSFs), offer distinct advantages over conventional linear models by capturing complex nonlinear interactions, particularly in rare malignancies with limited data sets.^9,10^ This study leverages the largest single-center ITT cohort to date, to our knowledge, to develop and internally validate an ML-based prognostic model. We integrated clinicopathologic parameters with immune-inflammatory markers to create a robust, web-based tool for personalized risk stratification.

## MATERIALS AND METHODS

### Study Population and Data Collection

**FIG 1.**
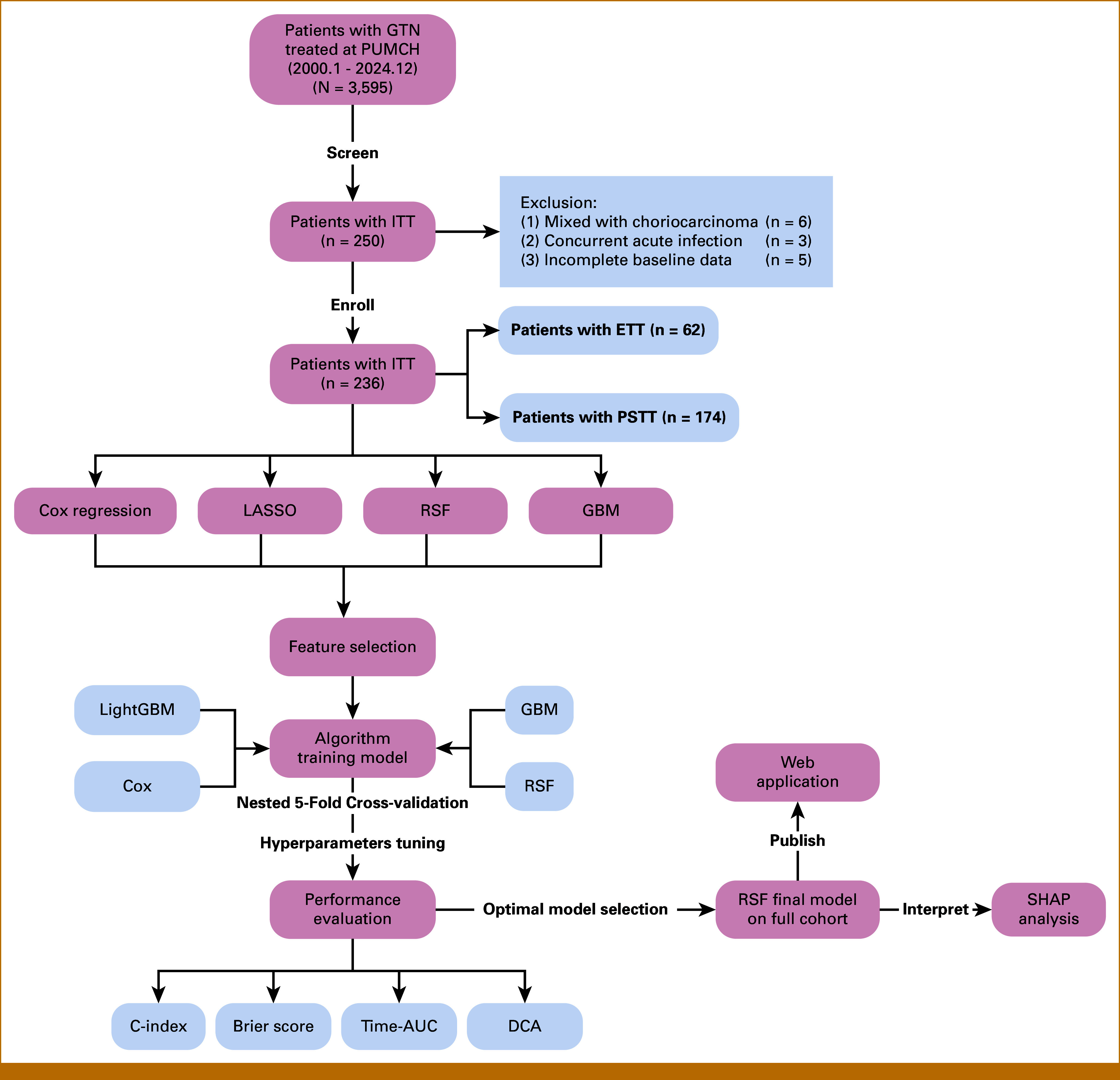
Study flowchart for model development and implementation. Cox, Cox proportional hazards regression; DCA, decision-curve analysis; ETT, epithelioid trophoblastic tumor; GBM, gradient boosting machine; GTN, gestational trophoblastic neoplasia; ITT, intermediate trophoblastic tumor; LASSO, least absolute shrinkage and selection operator; LightGBM, Light Gradient Boosting Machine; PSTT, placental site trophoblastic tumor; PUMCH, Peking Union Medical College Hospital; RSF, random survival forest; SHAP, SHapley Additive exPlanations.

We retrospectively screened 3,595 patients with GTN at Peking Union Medical College Hospital (2000-2024; Fig 1). A total of 236 patients (62 ETT, 174 PSTT) were included. Inclusion criteria required pathologically confirmed ITT and complete clinical data. Exclusion criteria were mixed choriocarcinoma histology, concurrent infection/autoimmune disease, or missing laboratory data. The study was approved by the PUMCH Ethics Committee (No. K3838) with a waiver for informed consent.

We collected demographic, clinical, and pathologic data, including age, antecedent pregnancy, pretreatment HCG, FIGO stage, Ki-67 index, and treatment record. Tumor burden was assessed using standardized CT/MRI. Pretreatment blood samples (1-2 weeks before therapy) yielded seven inflammatory and nutritional indices,^12^ including NLR, SIRI, PLR, MLR, SII, PIV, and PNI (formulas in Data Supplement, Table S1).

### Follow-Up and Outcome Assessment

Patients were followed until April 2025. Treatment responses were assessed using standardized criteria: complete remission (CR) defined as sustained normalization of serum HCG and radiologic resolution for ≥1 month; partial remission as a decline in HCG or tumor size without new lesions; progressive disease as rising HCG levels, enlarging lesions, or new metastases; and recurrence as disease reappearance after confirmed CR.

The primary end point was progression-free survival (PFS; treatment initiation to progression, relapse, or death). Secondary end points were overall survival (OS; treatment to death) and recurrence-free survival (RFS; CR to recurrence). For all survival analyses, time zero was defined as the date of the first ITT-directed therapy, specifically the date of primary surgery or first cycle of chemotherapy for patients receiving neoadjuvant/primary chemotherapy.

### Variable Selection and Model Development

In total, 61 PFS events among 236 patients were observed. We prespecified 20 clinicopathologic and inflammatory covariates on the basis of previous literature.^4,6,7,13-20^ (details in Data Supplement, Table S2). To ensure robust feature selection, we used an intersection strategy integrating four complementary approaches: (1) multivariable Cox regression, (2) LASSO regression (optimal λ by 5-fold cross-validation),^21^ (3) Gradient Boosting Machine (GBM) using SHapley Additive exPlanations (SHAP) importance,^22^ and (4) RSF using permutation importance.^23^ Only variables consistently selected across these methods were retained to minimize overfitting.

Four modeling approaches (Cox, RSF, GBM, and LightGBM)^24^ were applied to the final feature set. Hyperparameters were optimized using grid search coupled with internal resampling. We used a nested 5-fold cross-validation framework, strictly confining tuning to training folds to prevent data leakage. Performance was evaluated using pooled out-of-fold predictions, assessed by Harrell C-index, time-dependent AUC, Integrated Brier Score (IBS),^25,26^ and Brier Skill Score (BSS),^27^ with BSS at each horizon defined as follows:

$$\text{Brier}\;\text{Skill}\;\text{Score}\left(t\right)=1-\frac{\text{Brier}\;\text{Score}\left(\text{model}\right)\left(t\right)}{\text{Brier}\;\text{Score}\left(\text{ref}\right)}$$

where Brier Score(ref) = 0.25 is the Brier score of a noninformative forecast assigning an event probability of 0.5 to all patients. Clinical utility was determined using decision-curve analysis (DCA) within the actionable 0.20-0.60 threshold range. The optimal model was ultimately identified through a comprehensive assessment of discrimination, calibration, and clinical utility. For illustrative prognostic stratification, patients were then dichotomized into high-risk and low-risk groups according to the median predicted risk score derived from the final model.

### SHAP Analysis and Web-Based Tool Deployment

Following model selection, we used SHAP methodology^28^ to enhance model interpretability. This approach quantifies feature importance through unified value attribution, identifying key prognostic factors and visualizing their predictive contributions. The SHAP analysis revealed nonlinear variable-outcome relationships, providing clinicians with intuitive decision-support evidence through force plots and dependency graphs.

The optimized model was implemented as a clinician-facing web application using the Shinyapps platform. This interactive tool allows real-time input of patient characteristics to generate individualized risk predictions with corresponding survival curves.

### Statistical Analysis

Analyses were performed using R version 4.5.0 (Data Supplement, Table S3). Continuous variables were compared using Student t-test or Wilcoxon rank-sum test; categorical variables were compared using Chi-square or Fisher exact tests. Survival was estimated using the Kaplan-Meier method and log-rank test (two-tailed *P* < .05).

To facilitate clinical interpretation and risk stratification in descriptive Cox regression and Kaplan-Meier analyses, continuous variables were categorized as follows: Age was dichotomized at 40 years, pretreatment HCG was trichotomized (5 and 1,000 IU/L), and optimal cut points for other variables were determined using maximally selected rank statistics (surv_cutpoint). However, to ensure a completely fair and unbiased head-to-head comparison of predictive performance among all algorithms, the Cox proportional hazards model, RSF, GBM, and LightGBM were all trained and cross-validated using the exact same continuous predictors standardized through normalization.^22-24^ Sensitivity analyses were performed to assess temporal stability. The full analytical code for model development and validation is provided in Data Supplement (File S1; model).

## RESULTS

### Characteristics of Study Population

The study cohort comprised 236 patients with ITT, including 62 with ETT (26.3%) and 174 with PSTT (73.7%; Table 1). The median age was 32.5 years, and the median IFAP was 15 months. Disease was confined to the uterus (stage I) in 63% of patients, with a median pretreatment HCG of 114 IU/L. Patients with ETT exhibited significantly higher rates of pulmonary presentation (29% *v* 3%, *P* < .01) and lower uterine segment/cervical involvement (24% *v* 6%, *P* < .01) compared with those with PSTT, while maintaining comparable treatment modalities (*P* = .25) and first-line chemotherapy regimens (*P* = .11).

**TABLE 1.** Baseline Characteristics of Patients With ITT

Characteristic	ETT^a^ (n = 62)	PSTT^a^ (n = 174)	*P* ^b^	Total^a^ N = 236
Initial symptom			<.01	
Abnormal vaginal bleeding	33 (53%)	124 (71%)		157 (67%)
Amenorrhea	7 (11%)	23 (13%)		30 (13%)
Lower abdominal symptoms	12 (19%)	8 (5%)		20 (8%)
Respiratory symptoms	5 (8%)	2 (1%)		7 (3%)
Others^c^	5 (8%)	17 (10%)		22 (9%)
Age, years	34.8 (7.8)	31.6 (5.7)	<.01	32.5 (6.4)
FIGO stage			<.01	
I	22 (35%)	127 (73%)		149 (63%)
II	7 (11%)	2 (1%)		9 (4%)
III	23 (37%)	40 (23%)		63 (27%)
IV	10 (16%)	5 (3%)		15 (6%)
Antecedent pregnancy			.59	
Hydatidiform mole	4 (6%)	14 (8%)		18 (8%)
Induced abortion/miscarriage	16 (26%)	55 (32%)		71 (30%)
Full-term	42 (68%)	105 (60%)		147 (62%)
IFAP, months	33.5 (18.0, 74.3)	12.0 (7.0, 24.0)	<.01	15.0 (8.8, 33.0)
Pretreatment HCG, IU/L	282.7 (9.5, 1,221.0)	100.0 (11.7, 385.0)	.09	114.0 (11.5, 569.9)
Initial tumor site			<.01	
Uterus body	24 (39%)	157 (90%)		181 (77%)
Lower uterine segment/cervix	15 (24%)	10 (6%)		25 (11%)
Lungs	18 (29%)	6 (3%)		24 (10%)
Others^d^	5 (8%)	1 (1%)		6 (3%)
Maximum tumor size, cm	4.0 (2.1, 6.0)	3.5 (2.3, 4.9)	.23	3.5 (2.2, 5.0)
Number of lesions			<.01	
<3	43 (69.4%)	166 (95.4%)		209 (88.6%)
≥3	19 (30.6%)	8 (4.6%)		27 (11.4%)
Pulmonary metastasis	29 (47%)	44 (25%)	<.01	73 (31%)
Brain or liver metastasis	6 (10%)	4 (2%)	.02	10 (4%)
Distant metastasis to other organs^e^	10 (16%)	3 (2%)	<.01	13 (6%)
Treatment modality			.25	
Chemo only	1 (2%)	3 (2%)		4 (2%)
Surgery only	14 (23%)	59 (34%)		73 (31%)
Surgery + chemo	47 (76%)	112 (64%)		159 (67%)
First-line chemo regimen			.11	
No chemo	14 (23%)	59 (34%)		73 (31%)
EMA/CO	11 (18%)	34 (20%)		45 (19%)
FAEV/FAV	24 (39%)	64 (37%)		88 (37%)
EMA/EP	6 (10%)	5 (3%)		11 (5%)
Others^f^	7 (11%)	12 (7%)		19 (8%)
Myometrial invasion			.84	
<1/2	38 (61%)	111 (64%)		149 (63%)
≥1/2	24 (39%)	63 (36%)		87 (37%)
Ki-67 index	30.0 (15.0, 50.0)	15.0 (10.0, 26.8)	<.01	20.0 (10.0, 30.0)
NLR	3.0 (2.0, 4.1)	2.0 (1.6, 2.9)	<.01	2.3 (1.7, 3.1)
PLR	144.1 (107.8, 206.9)	123.3 (97.5, 152.3)	<.01	127.1 (99.8, 161.0)
MLR	0.2 (0.1, 0.3)	0.2 (0.1, 0.2)	.35	0.2 (0.1, 0.2)
SII	700.3 (405.6, 968.6)	403.5 (291.3, 606.2)	<.01	437.6 (327.2, 697.6)
SIRI	0.9 (0.5, 1.5)	0.6 (0.4, 0.9)	<.01	0.6 (0.4, 1.2)
PIV	192.9 (107.9, 337.5)	116.4 (81.3, 171.9)	<.01	126.9 (83.9, 219.3)
PNI	52.9 (49.5, 55.6)	54.0 (51.3, 57.3)	.03	53.6 (51.1, 56.8)

Abbreviations: EMA/CO, etoposide, methotrexate, actinomycin-D/cyclophosphamide, and vincristine; EMA/EP, etoposide, methotrexate, actinomycin-D/etoposide, and cisplatin; FAEV, 5-fluorouracil, actinomycin-D, etoposide, and vincristine; FAV, 5-Fluorouracil, actinomycin-D, and vincristine; FIGO, International Federation of Gynecology and Obstetrics; HCG, human chorionic gonadotropin; IFAP, interval from antecedent pregnancy; ITT, intermediate trophoblastic tumor; MLR, monocyte-to-lymphocyte ratio; NLR, neutrophil-to-lymphocyte ratio; PIV, pan‑immune-inflammation value; PLR, platelet-to-lymphocyte ratio; PNI, prognostic nutritional index; SD, standard deviation; SII, systemic immune-inflammation index; SIRI, systemic inflammation response index.

^a^ n (%); mean (SD); median (IQR).

^b^ Statistical tests used for comparisons between ETT and PSTT were as follows: (1) Student t-test—age; (2) Wilcoxon rank-sum test—IFAP, pretreatment HCG, maximum tumor size, Ki-67 index, NLR, PLR, MLR, SII, SIRI, PIV, and PNI; (3) Pearson Chi-square test—initial symptom, FIGO, stage, antecedent pregnancy, initial tumor site, number of lesions, pulmonary metastasis, treatment modality, first-line chemotherapy regimen, and myometrial invasion; (4) Fisher exact test—brain or liver metastasis and distant metastasis to other organs.

^c^ Including vaginal discharge (n = 1), vaginal mass (n = 3), incidentally found on imaging during physical examination (n = 5), early pregnancy symptoms (n = 3), and elevated blood HCG levels only (n = 10).

^d^ Including cesarean section scar tissue (n = 2), vagina (n = 2), ovary (n = 1), and rectouterine pouch (n = 1).

^e^ Including the intestines (n = 5), pancreas (n = 3), adrenal glands (n = 1), kidneys (n = 3), bladder (n = 3), or spleen (n = 1) metastasis.

^f^ Including TP/TE, paclitaxel, cisplatin/paclitaxel, and etoposide (n = 3); BEP, bleomycin, etoposide, and cisplatin (n = 2); FA = 5-Fluorouracil and actinomycin-D (n = 2); 5-FU (5-Fluorouracil) only (n = 5); MTX (methotrexate) only (n = 3); and KSM (actinomycin-D) only (n = 4).

### Treatment and Outcomes

The cohort included 149 patients with stage I (63%) and 87 with stage II-IV (37%) ITT (Data Supplement, Table S4; Fig S1). Surgical resection was performed in 98.3% (232/236) of all patients. Combined surgery-chemotherapy was significantly more frequent in stage II-IV than stage I patients (88.5% *v* 55.0%, *P* < .01), as were chemotherapy intensity and incomplete remission rates (CR, 73.6% *v* 99.3%, *P* < .01). First-line chemotherapy regimens showed no intergroup difference (*P* = .73).

With a median follow-up of 52.8 months, prognosis was strongly stage dependent (*P* < .01; Data Supplement, Fig S2A-F). Stage I patients achieved 100% 5-year OS, with 94% 3-year RFS (95% CI, 89.8 to 98.4) and 87.1% PFS (95% CI, 81.5 to 93.1). By contrast, stage II-IV patients had a 5-year OS of 82.1% (95% CI, 73.2 to 92.0), 3-year RFS of 63.5% (95% CI, 52.1 to 77.3), and PFS of 46.6% (95% CI, 36.4 to 59.6). Stage IV patients exhibited a particularly dismal prognosis with 5-year OS plunging to 26.4% (95% CI, 10.4 to 66.8), median OS of 36 months, and median PFS of merely 6.1 months.

Beyond FIGO staging, survival analysis identified four additional significant predictors of reduced PFS (all *P* < .01; Data Supplement, Fig S3A-D): IFAP ≥24 months, Ki-67 index ≥25%, NLR ≥2.5, and SIRI ≥1.1. However, survival outcomes showed no significant difference among various first-line chemotherapy regimens (*P* = .392; Data Supplement, Fig S3E).

### Prognostic Factor Identification

**FIG 2.**
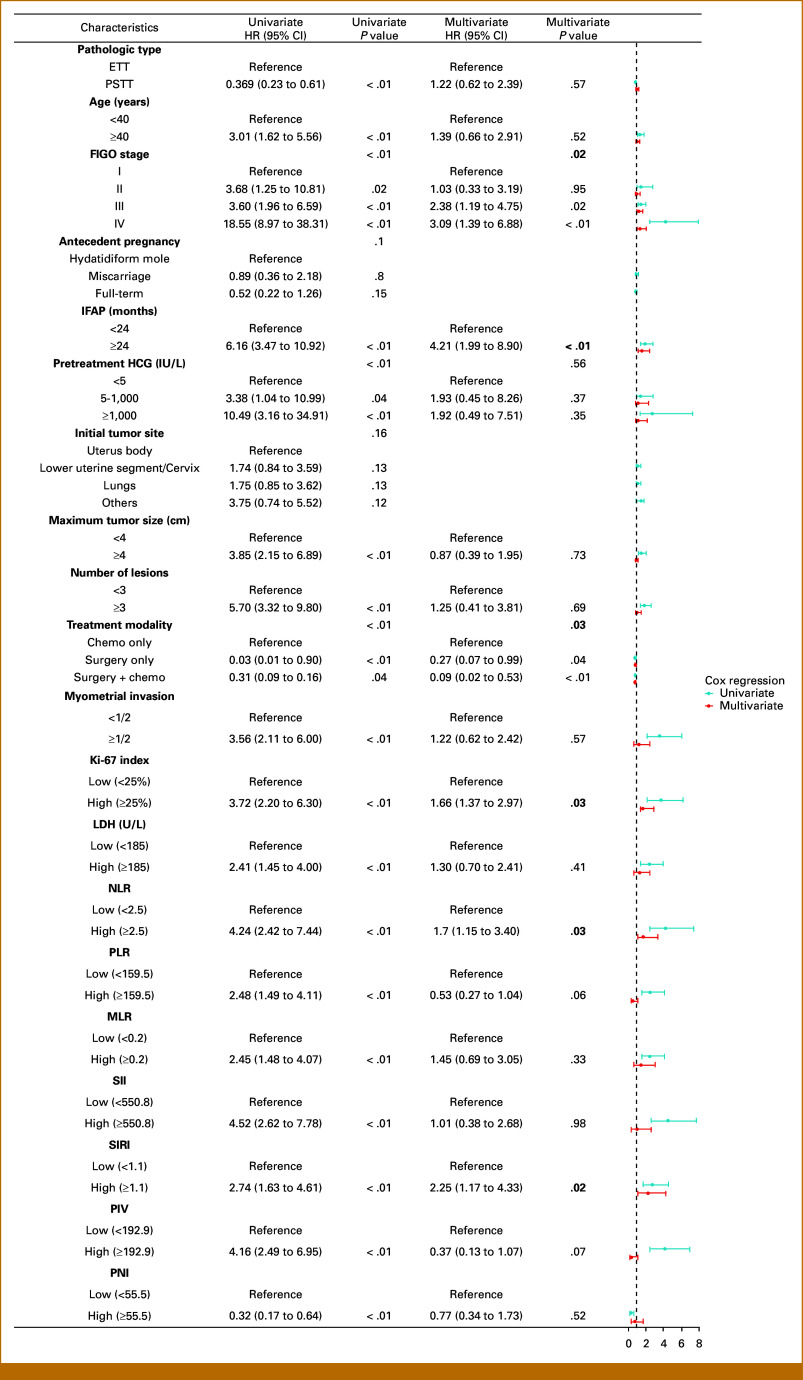
Forest plot showing univariate and multivariate Cox regression analyses for PFS in patients with ITT. HRs and 95% CIs are presented for each variable. ETT, epithelioid trophoblastic tumor; HCG, human chorionic gonadotropin; HRs, hazard ratios; IFAP, interval from antecedent pregnancy; ITT, intermediate trophoblastic tumor; LDH, lactate dehydrogenase; MLR, monocyte-to-lymphocyte ratio; NLR, neutrophil-to-lymphocyte ratio; PFS, progression-free survival; PIV, pan‑immune-inflammation value; PLR, platelet-to-lymphocyte ratio; PNI, prognostic nutritional index; PSTT, placental site trophoblastic tumor; SII, systemic immune-inflammation index; SIRI, systemic inflammation response index.

**FIG 3.**
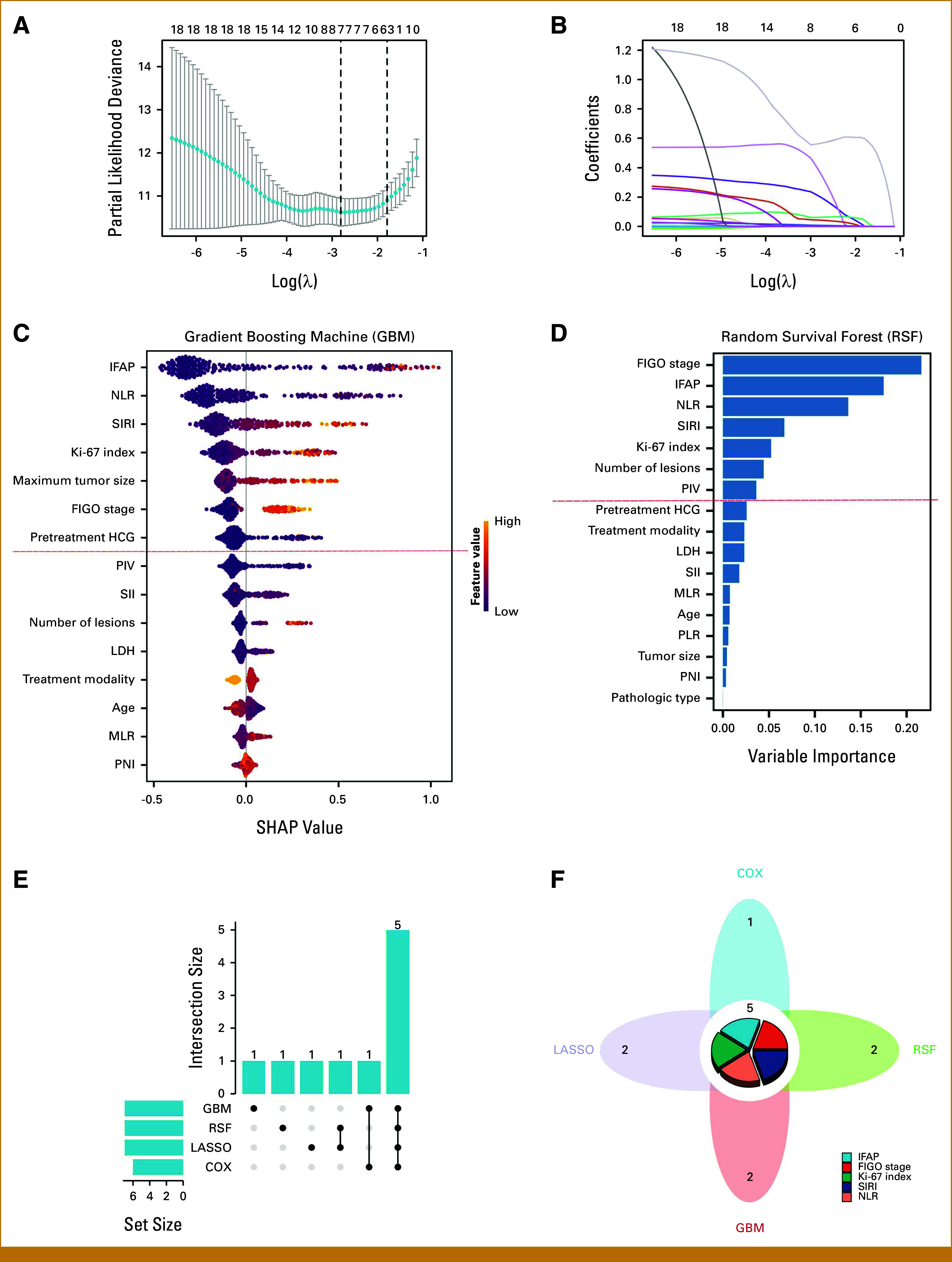
Feature selection using four ML methods. (A) Partial likelihood deviance plot from LASSO regression for optimal lambda selection. (B) LASSO coefficient profiles for candidate variables. (C) SHAP summary plot showing feature contributions in the GBM model. (D) Variable importance ranking from the RSF model. (E) Upset plot showing overlap of selected features among the four methods. (F) Venn diagram illustrating the five intersecting features selected by all algorithms. GBM, gradient boosting machine; LASSO, least absolute shrinkage and selection operator; ML, machine learning; RSF, random survival forest; SHAP, SHapley Additive exPlanations.

During follow-up, 61 PFS events were recorded. Initial univariable screening identified 18 candidate variables associated with PFS (*P* < .05; Fig 2). To ensure robust feature selection, we used four complementary approaches (detailed in Supplementary Table S2): Multivariable Cox regression identified six predictors, with FIGO stage IV (hazard ratio [HR], 3.09, *P* < .01) and IFAP ≥24 months (HR, 4.21, *P* < .01) showing the strongest associations (Fig 2). LASSO selected seven variables (Figs 3A and 3B). GBM and RSF ranked features by SHAP and permutation importance, respectively (Figs 3C and 3D). Intersecting these four methods yielded a stable consensus of five predictors: FIGO stage, IFAP, Ki-67 index, NLR, and SIRI (Figs 3E and 3F). This rigorous selection process ensured that the final model used only the most consistent prognostic signals across linear and nonlinear frameworks.

Sensitivity analyses confirmed the temporal stability of this prognostic signature. Baseline characteristics and survival outcomes remained consistent across three calendar eras (2000-2024; Data Supplement, Table S5A, Fig S4). Furthermore, year of diagnosis was not a significant predictor in multivariable analysis (*P* = .35; Data Supplement, Table S5B), indicating that the model’s performance was robust to historical changes in clinical practice.

### Model Development and Performance Evaluation

**FIG 4.**
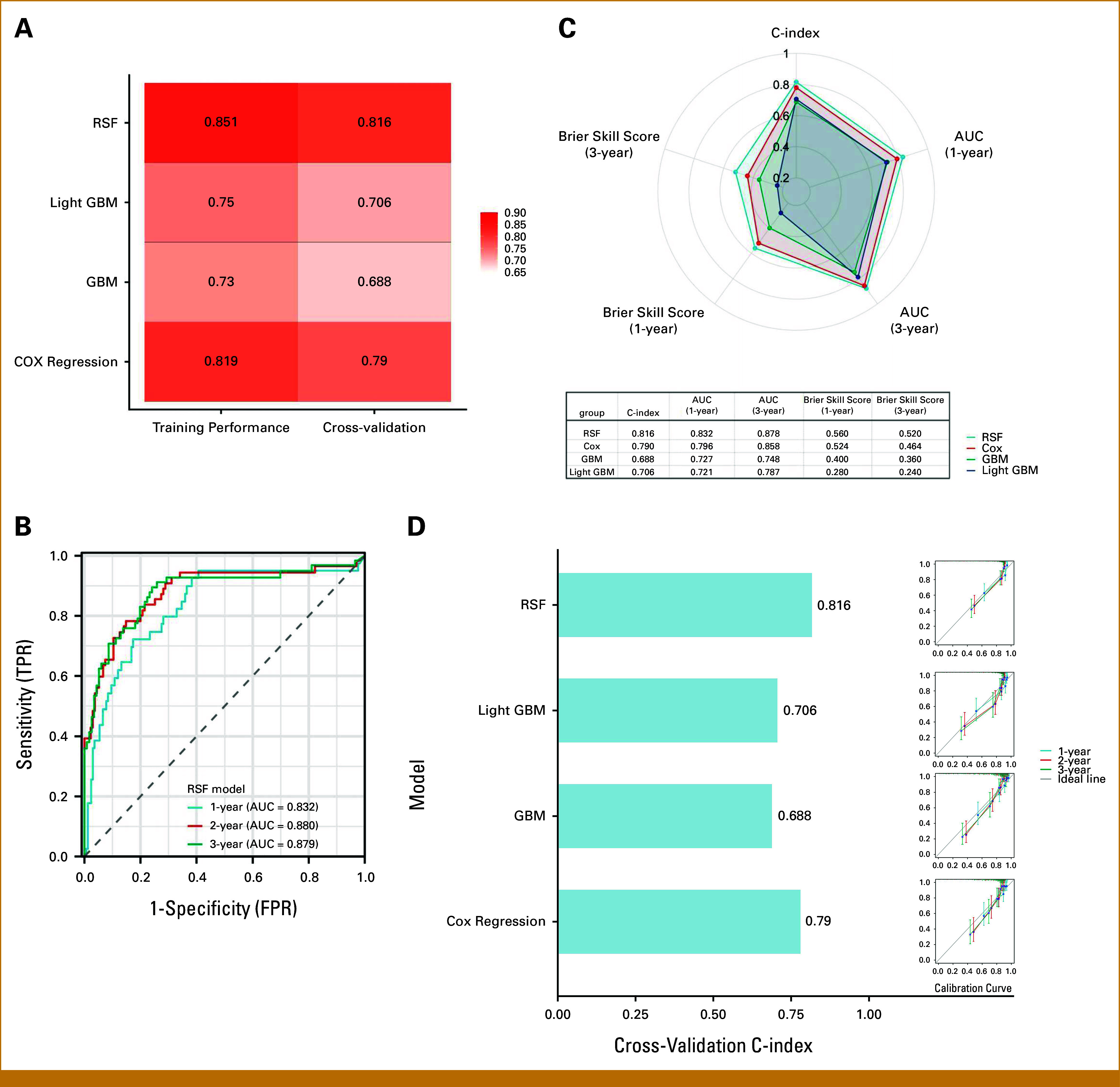
Model performance comparison among four prognostic algorithms for PFS in patients with ITT. (A) Heatmap of C-index values for training and cross-validation performance. (B) Time-dependent ROC curves and AUC values for the RSF model at 1-, 2-, and 3-year time points. (C) Radar plot comparing C-index, AUC, and BSS across four models. Brier Skill Score(t) = 1 - [Brier Score_(model)_ (t)/Brier Score_(ref)_], where Brier Score_(ref)_ = 0.25 is the Brier score of a noninformative forecast assigning an event probability of 0.5 to all patients. Thus, BSS(t) = 0 corresponds to a model with no skill relative to this reference, and BSS(t) = 1 indicates perfect prediction. Corresponding Brier scores are provided in the Data Supplement (Table S10). (D) Bar plot of cross-validated C-index with corresponding 1-, 2-, and 3-year calibration curves for each model. BSS, Brier Skill Scores; FPR, false-positive rate; GBM, gradient boosting machine; ITT, intermediate trophoblastic tumor; LightGBM, Light Gradient Boosting Machine; PFS, predicting progression-free survival; ROC, receiver operating characteristic; RSF, random survival forest; TPR, true-positive rate.

Using the five selected prognostic variables, we developed PFS prediction models through four approaches: Cox regression, RSF, GBM and LightGBM (final model details in Data Supplement, Tables S6-S9). We evaluated the four prognostic models using a rigorous nested 5-fold cross-validation framework, ensuring that hyperparameter tuning was strictly isolated from performance assessment (Data Supplement, Table S10). The RSF model demonstrated superior discrimination, achieving the highest cross-validated C-index of 0.816 (95% CI, 0.721 to 0.895), significantly outperforming the continuous Cox (0.790), LightGBM (0.706), and GBM (0.688) models (Fig 4A). Temporally, the RSF model maintained robust predictive accuracy, with 1- and 3-year time-dependent AUCs of 0.832 and 0.879, respectively (Fig 4B). Furthermore, it exhibited the best overall calibration, indicated by the highest BSS (Fig 4C) and the lowest IBS (0.113). Calibration plots confirmed excellent agreement between predicted and observed survival probabilities (Fig 4D).

Clinical DCA confirmed the RSF model’s utility. It consistently yielded higher net benefit than “treat-all,” “treat-none,” or alternative models within the clinically relevant threshold probability range of .20-.60 at 1, 2, and 3 years (Data Supplement, Fig S5). Uncertainty analysis using 1,000 bootstrap resamples confirmed the stability of these net benefit estimates (Data Supplement, Table S11). Using the median RSF risk score (3.7 points) as a stratification cutoff, the model effectively dichotomized patients into low-risk and high-risk groups (Data Supplement, Fig S6). Specifically, the high-risk group exhibited observed 1-, 2-, and 3-year progression probabilities of 35%, 51%, and 57%, respectively (Data Supplement, Fig S5D). These values align precisely with the mid-to-upper spectrum of the 0.20-0.60 threshold range, validating this interval as a critical zone for therapeutic decision making. Such distinct risk stratification underscores the model’s capacity to identify candidates for intensified therapy while sparing lower risk patients from unnecessary intervention.

Sensitivity analyses further validated the model’s robustness. The full RSF model significantly outperformed a FIGO stage–only model, improving the C-index from 0.647 to 0.816 (Data Supplement, Table S12A). Importantly, the RSF model retained strong discriminative ability in both the stage I (C-index, 0.840) and stage II-IV (C-index, 0.801) subgroups (Data Supplement, Table S12B). Exploratory analyses also showed that the RSF score could stratify risk within specific treatment modalities, identifying high-risk patients even among those treated with surgery alone (Data Supplement, Fig S7).

### Model Interpretation and Prediction Tool Implementation

**FIG 5.**
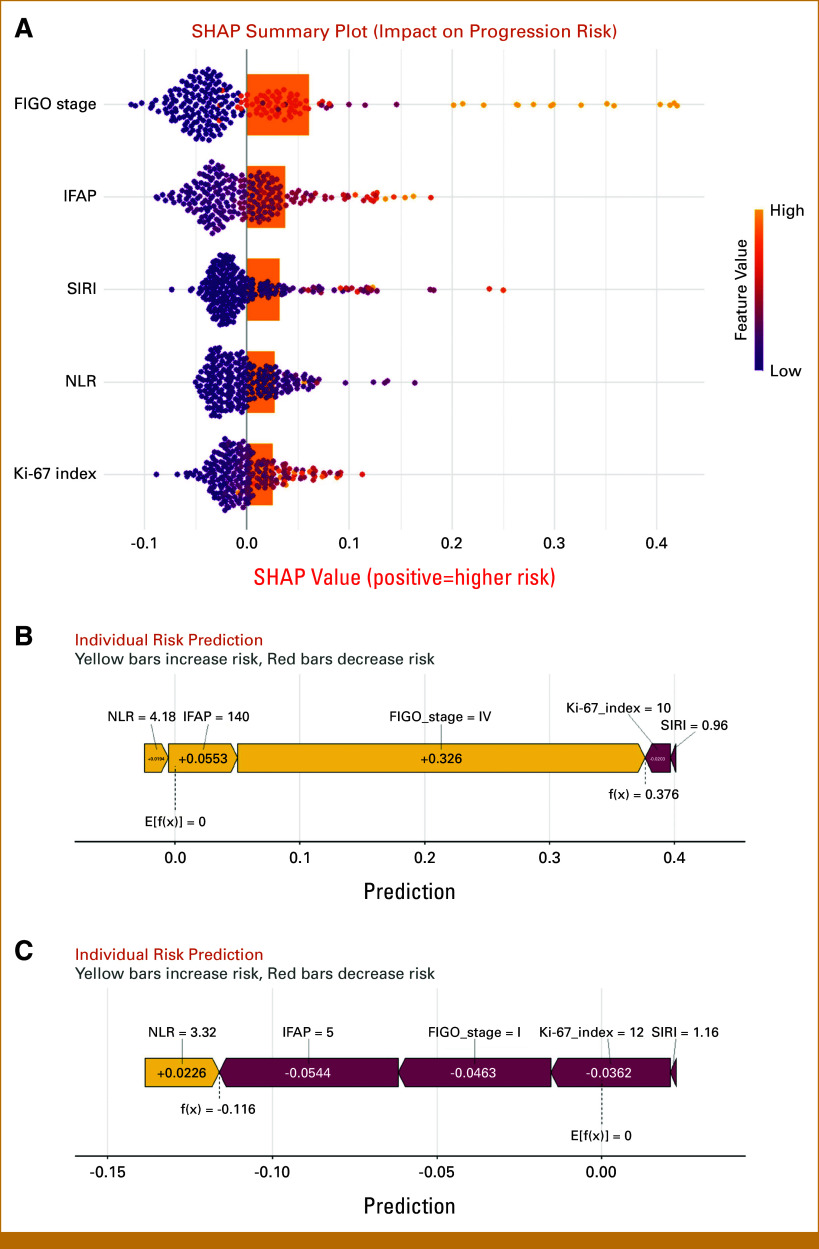
SHAP-based interpretation of the RSF model. (A) SHAP summary plot showing the overall contribution of each feature to progression risk; colors represent feature values (purple = low, yellow = high). (B) Force plot for a representative high-risk patient case, with cumulative positive SHAP contributions pushing risk upward. (C) Force plot for a representative low-risk patient case, where SHAP values are primarily negative, indicating protective effects. FIGO, International Federation of Gynecology and Obstetrics; IFAP, interval from antecedent pregnancy; NLR, neutrophil-to-lymphocyte ratio; RSF, random survival forest; SHAP, SHapley Additive exPlanations; SIRI, systemic inflammation response index.

SHAP analysis illuminated the RSF model’s decision-making logic. Globally, FIGO stage and IFAP emerged as the dominant risk drivers (Fig 5A). Dependency plots revealed nonlinear, synergistic interactions where advanced stage amplified the risk associated with inflammatory markers (Data Supplement, Fig S8). At the individual level, force plots transparently visualized how specific variable combinations—such as advanced stage coupled with high NLR—cumulatively elevate progression risk or, conversely, how favorable profiles confer protection (Figs 5B and 5C).

To facilitate clinical application, we deployed an interactive web tool^29^ (Data Supplement, Fig S9). This platform autocalculates inflammatory indices and generates real-time, individualized PFS predictions. Key features include (1) dynamic survival curves with 95% CIs to visualize uncertainty, (2) explicit calculation of cumulative progression risk (1-PFS), and (3) a strict 5-year prediction horizon to prevent unreliable long-term extrapolation. This implementation transforms complex ML outputs into actionable, transparent clinical insights. Crucially, acknowledging the potential for overoptimistic performance estimates because of selection bias in the training cohort, the web application currently functions strictly as a research and demonstration prototype. A prominent disclaimer has been embedded in the tool to explicitly prohibit its use for real-world clinical decision making until external validation is achieved.

## DISCUSSION

This study establishes the first ML-based prognostic model for ITT using the largest single-center cohort reported. By integrating FIGO stage, IFAP, Ki-67, NLR, and SIRI through a rigorous nested cross-validation framework, the RSF model (C-index, 0.816) outperformed anatomic staging and other alternative models. The deployment of an interpretable web tool translates these findings into actionable clinical utility.

Our cohort, derived from the largest national GTN referral center (3,595 patients over 24 years), showed a higher prevalence of ITT (6.9%) than historical estimates (1%-3%),^1,30^ likely reflecting referral bias. Key clinical features, including antecedent term pregnancy (62%) and abnormal bleeding (67%), aligned with previous reports.^1,13^ Notably, we observed distinct metastatic patterns: Pulmonary involvement was significantly more frequent in ETT than PSTT (29% *v* 5%; *P* < .01), consistent with recent findings on pulmonary-isolated GTN.^18^ Our cohort demonstrated a lower median pretreatment HCG (114 IU/L) compared with some historical series (approximately 15-665 IU/L),^7,13-15^ likely reflecting earlier diagnosis and active surveillance. Notably, 18.2% of patients had undetectable baseline HCG, limiting its utility as a sole prognosticator. Consistent with previous findings,^13,15^ HCG failed to independently predict PFS in multivariable analysis.

Surgery remains the cornerstone of ITT management.^4,5,31^ Our cohort reflects a surgery-oriented practice, with 98.3% of patients undergoing resection. This aggressive surgical approach, coupled with a high proportion of early-stage disease (63%), likely contributes to the observed low mortality rate (5.5%), which aligns with large-scale Chinese data (6.5%)^13^ but is lower than Western series (approximately 20%).^4,7,15^ Stage I patients treated with surgery alone achieved excellent outcomes, whereas adjuvant chemotherapy provided no PFS benefit in unselected patient cases (*P* < .01; Data Supplement, Fig S10A), suggesting potential overtreatment. The RSF model corroborated this, assigning significantly lower risk scores to surgery-only patients (Data Supplement, Fig S10B), indicating that adjuvant therapy could be selectively reserved for high-risk individuals.

For advanced disease, although combined surgery-chemotherapy is standard, we found no significant PFS difference among diverse first-line regimens (FAEV/FAV, EMA/CO, EMA/EP; *P* = .392). This real-world evidence supports flexibility in regimen selection, potentially favoring 5-Fluorouracil‑based protocols. Notably, a subset of carefully selected stage II-III patients treated with surgery alone achieved outcomes comparable with those receiving combined therapy (Data Supplement, Fig S10C,D). Although exploratory, these findings, supported by favorable RSF risk trends (Data Supplement, Fig S10B), hint at the possibility of de-escalating treatment in low-risk advanced patient cases. Aligning with surgical guidelines,^1-3^ our model exclusively uses pretreatment factors to serve as a baseline risk stratification tool.

Consistent with international consensus,^3,13,15,30^ FIGO stage IV remains the most powerful independent prognostic factor (HR, 3.09). The dismal 5-year OS of 26.4% in our stage IV cohort highlights the severe disease burden in this subgroup, which was accurately captured by the RSF model (mean risk score = 47.8). This identification of ultra‑high-risk patients is critical for guiding determining candidates for intensified therapies, including emerging immunotherapies.^3^

While IFAP ≥48 months is the standard overall survival threshold,^4,7,15^ ≥24 months optimally predicted progression (HR, 4.21). This variation reflects the distinct clinical end points: A shorter interval is more sensitive for stratifying early progression/recurrence risk, suggesting outcome-specific thresholds could better guide surveillance intensity.

We also provide novel evidence, to our knowledge, for biologic markers. Ki-67, a well-established proliferation marker in epithelial malignancies,^32^ is traditionally used for diagnostic differentiation in trophoblastic disease.^30^ In this study, we demonstrate for the first time, to our knowledge, that high Ki-67 is an independent predictor of worse PFS in ITT, though its widespread clinical utility remains limited by interobserver variability in manual counting^33^ and sampling bias from tumor heterogeneity; larger validation studies incorporating digital image analysis are warranted. Furthermore, we established NLR and SIRI as independent prognostic indicators in ITT, aligning with emerging evidence in other cancers.^9,10^ These markers provide a comprehensive snapshot of the host-tumor immune balance: Neutrophils promote tumor progression through reactive oxygen species and neutrophil extracellular traps,^34^ while chronic inflammation contributes to treatment resistance.^8^ Conversely, lymphocyte-mediated responses exhibit antitumor effects.^35^ The unique invasiveness and immunogenicity of trophoblastic tumors^1^ underscore the relevance of this systemic inflammatory status. Although previous studies linked NLR to choriocarcinoma chemoresistance,^19,20^ our findings extend this to ITT, offering cost-effective tools for individualized risk stratification.

This study advances ITT prognostic modeling by overcoming key limitations of previous SEER-based efforts.^36^ We incorporated critical clinical variables often missing in public databases (eg, antecedent pregnancy history), ensured central pathologic review to minimize diagnostic bias, and developed the model exclusively for ITT to guarantee disease specificity. Methodologically, our approach introduced three innovations: (1) using time-to-event modeling rather than binary classification to align with clinical reality, (2) using SHAP analysis to decode complex feature contributions, and (3) validating clinical utility using DCA. Furthermore, the stability of the final model was corroborated by internal validation using out-of-bag estimates (Data Supplement, Fig S11)

Rather than prescribing a rigid universal cutoff, we identified a clinically relevant probability range of 0.20-0.60 as the zone of equipoise, where treatment intensification decisions are most critical. In our cohort, risks less than 20% rarely warrant escalation beyond standard surgery with or without conventional chemotherapy, whereas risks more than 60% almost invariably trigger aggressive therapy. Within this intermediate window, the RSF model demonstrated superior net benefit. To operationalize these findings, we deployed the first web-based ITT prediction tool.^29^ By generating individualized survival probabilities and dynamic curves, this tool facilitates risk-stratified management: supporting treatment de-escalation for low-risk patients to optimize quality of life, while guiding intensified therapy and surveillance for high-risk patients.

We acknowledge several limitations. First, despite temporal stability in sensitivity analyses (Data Supplement, Table S5), unmeasured heterogeneity across the 24-year time frame cannot be entirely excluded. Second, integrating molecular biomarkers using deep learning^37^ in larger cohorts remains a future goal to enhance predictive accuracy. Third, initial feature selection on the full cohort introduces potential selection bias and residual optimism. We accepted this compromise because fold-wise variable selection on a small data set yields unstable feature sets, precluding a deployable model. Consequently, the web tool is restricted to “research use only” pending external validation. Finally, our surgery-oriented, Asian tertiary center cohort may limit generalizability regarding treatment patterns and demographics. As ITT is relatively chemoresistant and surgery is the mainstay of treatment, our model is primarily applicable to surgically managed patients; external validation in diverse cohorts is essential to confirm its broader applicability.

In conclusion, we developed and internally validated the first ML-based prognostic model specifically for ITT. By integrating five robust predictors—including novel immune-inflammatory markers—the RSF model achieves superior accuracy compared with traditional staging or alternative models. We successfully translated this algorithm into a user-friendly, web-based tool that provides real-time, individualized risk stratification. Although current performance metrics may be optimistic and require independent external validation, these findings provide a foundational framework supporting a paradigm shift toward precise, risk-adapted management for ITT. Future multicenter prospective studies incorporating molecular profiling are warranted to further validate and expand the utility of this tool across diverse populations.

## Data Availability

A data sharing statement provided by the authors is available with this article at DOI https://doi.org/10.1200/PO-25-00881. Data are available on reasonable request. The corresponding author could be contacted with requests.
